# Supplementary material for: Laboratory realization of relativistic pair-plasma beams
Source: Nat Commun. 2024 Jun 12;15:5029. doi: 10.1038/s41467-024-49346-2 (PMC11169600; doi:10.1038/s41467-024-49346-2)
Supplement: Supplementary file 1 — Supplementary Information [file 41467_2024_49346_MOESM1_ESM.pdf]

Laboratory realization of relativistic pair-plasma beams

Supplementary information

1 Detailed secondary beam characteristics 1

2 Beam elongation due to particle straggling 2

3 Chromox luminescence as a function of particle type and energy 2

4 Processing the raw data from the Chromox screen images 3

5 Fitting relativistic distribution functions to the $e^{\pm}$ pair population 4

6 Parameter scan of target material thicknesses using FLUKA simulations 6

1 Detailed secondary beam characteristics

Characteristics of the secondary beam components at the rear surface of the target are obtained from a FLUKA simulation (Supplementary Table 1). Particle spectra and emittance are shown in Supplementary Figure 1.

Species	Specified energy range	Yield (per primary)	Mean energy, $\langle E \rangle$ (GeV)	Beam radius, σ_r (mm)
e^-	$\geq$ 1 MeV	56	0.20	2.0
e^+	$\geq$ 1 MeV	46	0.27	1.9
p^+	$\geq$ 100 keV, $<$ 430 GeV	1.4	31	1.2
p^+	$\geq$ 430 GeV	0.42	439	1.2
$\pi^{\pm}$	$\geq$ 100 keV	6.2	6.6	1.7
$k^{\pm}$	$\geq$ 100 keV	0.5	11	1.4
μ^-	$\geq$ 100 keV	<0.05	-	-
γ	$\geq$ 10 keV	600	0.07	2.0
n	$\geq$ 100 keV	12.6	3.1	2.2

Supplementary Table 1: Secondary beam composition from FLUKA simulations. The yield (per primary), mean energy and beam radius (half-width-half-maximum) are provided for the most-abundantly produced secondary species: electrons (e^-), positrons (e^+), protons (p^+), pions ($\pi^{\pm}$), kaons ($k^{\pm}$), muons (μ^-), γ-rays and neutrons (n). In the case of electrons and positrons, for ease of comparison with other studies, the low-energy cutoff is 1 MeV, though there are even more pairs (up to 10% more) if the energy cutoff is reduced to 10 keV. For the remaining species, the energy cutoff is defined by the simulation.

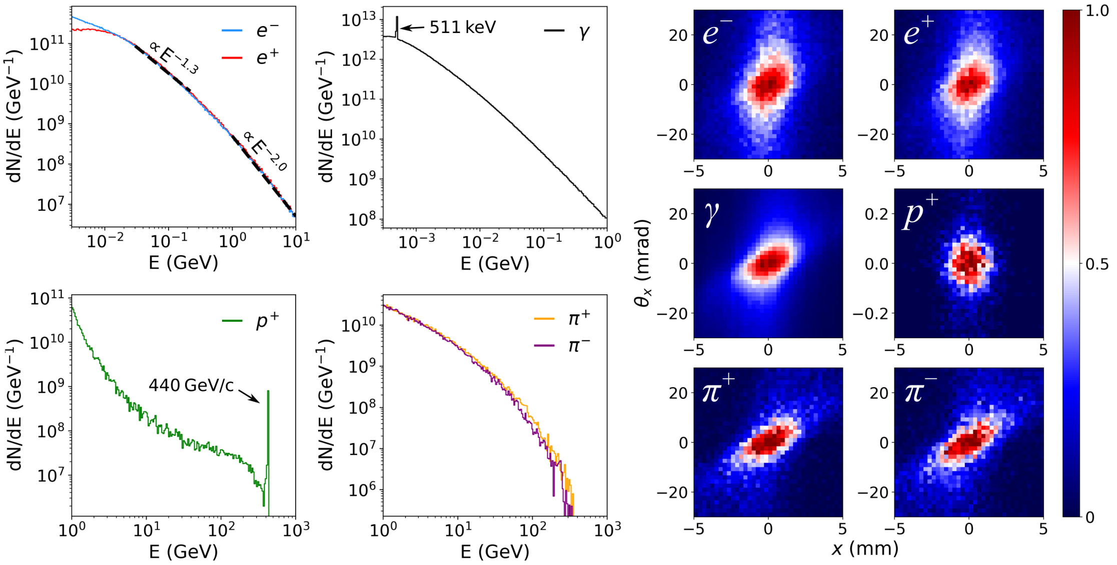

Supplementary Fig. 1: Secondary beam characteristics obtained from FLUKA simulations. (Adapted from Arrowsmith, C. D. et al. (2021) [1] in accordance with reuse and permission guidelines, assuming the exact SPS beam conditions and target configuration used in the experiment.) Energy spectra (left) and angle-position phase space (emittance) plots (right) at the immediate rear of the target. $e^{\pm}$ have a multi-power-law spectra, with spectral index $dN/dE \propto E^{-1.3}$ in the $30 - 220$ MeV range, and $dN/dE \propto E^{-2.0}$ at $E > 1$ GeV. In the emittance plots, x is the direction transverse to beam propagation and $\theta_x = \arctan(v_x/v_z)$ is the pointing angle of the velocity vector relative to the beam axis.

2 Beam elongation due to particle straggling

The longitudinal beam profile of the $e^{\pm}$ pairs is calculated using FLUKA simulations accounting for the particle straggling (the spread of times that particles breakthrough from the rear of the target). Supplementary Figure 2 shows that straggling time of $e^{\pm}$ pairs is $\lesssim 5\,\mathrm{ps}$, which is much smaller than the duration of the primary beam ($\sigma_t = 250\,\mathrm{ps}$) and justifies the assumption of an identical profile.

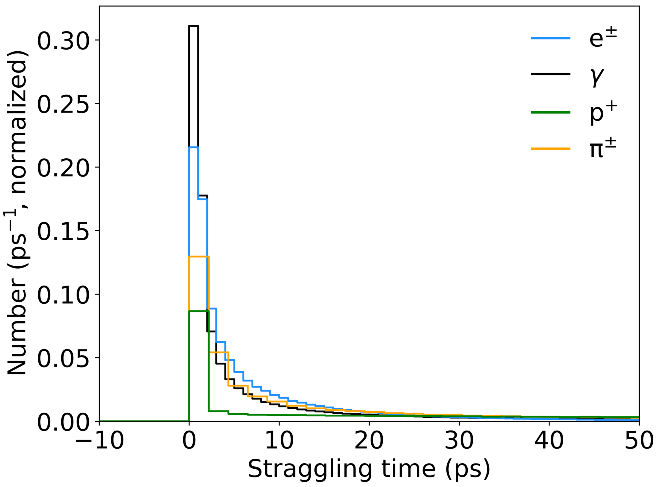

Supplementary Fig. 2: **FLUKA simulations of the straggling time of secondary species.** The distributions of times when particles breakthrough the rear surface of the target is obtained for different secondary species using a FLUKA simulation. The species plotted are; electrons/positrons ($e^{\pm}$), γ-rays, protons (p^{+}) and charged pions ($\pi^{\pm}$). The time $t = 0$ represents the earliest possible arrival time of particles at the rear of the target assuming they experience zero straggling. The straggling times are much smaller than the pulse duration of the primary beam.

3 Chromox luminescence as a function of particle type and energy

To validate the assumption that particles deposit an approximately equal amount of energy into the screens, FLUKA simulations are performed, irradiating Chromox with different particle types and energies. The results are shown in Supplementary Figure 3. In the range $2 \lesssim \Gamma \lesssim 500$, the deposited energy per particle is approximately the same and irrespective of the particle species. The energy deposited per γ-ray in the simulated range $50\,\mathrm{keV} - 1\,\mathrm{TeV}$ is also approximately constant (always within the range represented by the black shaded area), but it is $> 100\times$ smaller so the contribution of γ-rays in observed screen luminescence is expected to be negligible.

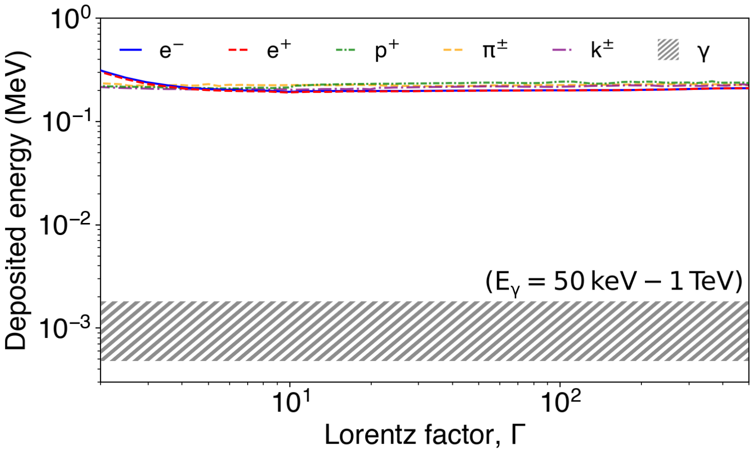

Supplementary Fig. 3: **FLUKA simulations of energy deposited into Chromox screens.** The energy deposited per particle into a 250 µm-thickness Chromox screen at 45° incidence is obtained from FLUKA simulations. Deposited energy is plotted as a function of relativistic Lorentz factor for: e^{-} (blue-solid), e^{+} (red-dashed), p^{+} (green-dot-dashed), $\pi^{\pm}$ (orange-dashed) and $k^{\pm}$ (purple-dot-dashed). The energy deposited per γ-ray in the simulated range $50\,\mathrm{keV} - 1\,\mathrm{TeV}$ is also approximately constant (always within the range represented by the black shaded area).

4 Processing the raw data from the Chromox screen images

The most significant source of background in images of the Chromox screens is direct irradiation of the camera sensor by high-energy hadrons and other radiation scattered around the experimental area, leading to a uniformly-distributed speckle noise that scales linearly with primary proton intensity and is more pronounced when the target is irradiated. In the analysis of the data, an intensity-dependent zero-level is established for the images by fitting the average noise level in regions of the images outside of the luminescence screen (shown in Supplementary Figure 4).

To process the images of the transverse beam profile, the zero-level is set using the fit to the intensity-dependent background, and images from multiple shots are combined. No median filtering is applied because combining multiple images naturally eliminates noise without distorting the underlying distribution. The same subtraction procedure is applied to the raw image data of the screens in the magnetic particle spectrometer (shown in Supplementary Figure 5). The increase in signal when the electromagnet is turned on is evidence of positrons in the beam.

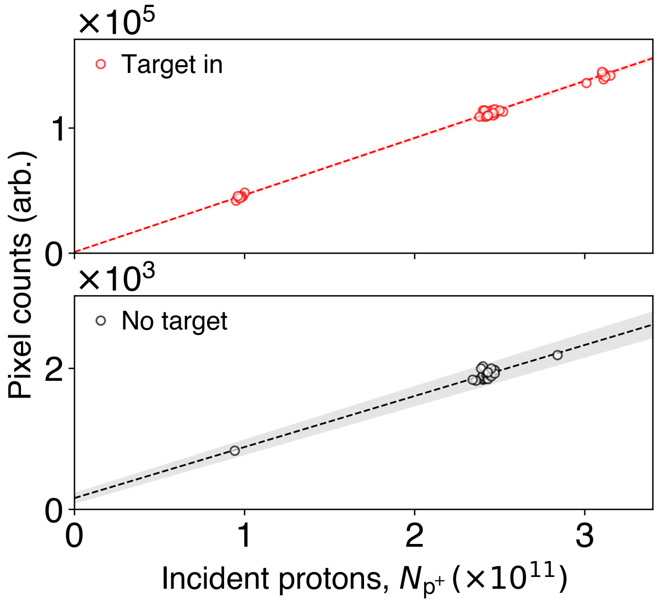

Supplementary Fig. 4: **Background pixel counts providing a zero-level in the luminescence screen images.** The mean pixel count is calculated in the image region outside of the luminescence screens to account for spurious signals. The top panel shows the mean pixel counts when the target is irradiated, and the bottom panel shows when the target is removed. In both cases, the background level is linearly proportional to the proton intensity. Linear fits of the mean pixel count are used to define a zero-level for the image intensity. The shaded regions correspond to the standard errors of the linear fit.

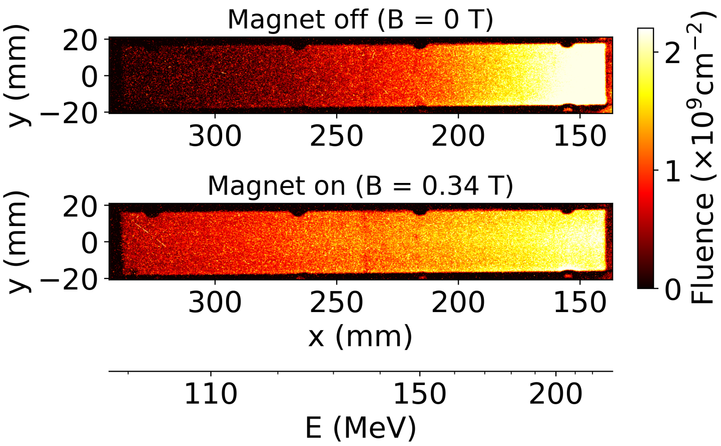

Supplementary Fig. 5: **Magnetic particle spectrometer raw data (positron screen).** Raw image data of the luminescence screens used to measure the energy spectra of electrons and positrons. The screen corresponding to positrons is shown when the target is irradiated and the electromagnet is turned off (upper), and when the electromagnet is turned on with peak magnetic field $B = 0.34\,\mathrm{T}$ (lower). When the magnet is turned on, the luminescence is enhanced. The secondary x-axis shows the corresponding positron energy according to the energy calibration. To construct the spectra, a vertical line-out is taken from the central 25 mm of the image.

5 Fitting relativistic distribution functions to the $e^{\pm}$ pair population

Alternative approaches of comparing relativistic electron-positron pair beam dimensions with the Debye screening length and the collisionless plasma skin depth consider the equivalent non-relativistic expressions and adjust the plasma density according to the mean Lorentz factor of the distribution. In effect, a cold non-relativistic thermal spread is assumed but for a population moving with a bulk velocity according to the mean Lorentz factor. However, this ignores the effect of the relativistic thermal spread in the screening. In addition, for non-thermal distributions the mean Lorentz factor often depends on the range of energies included in the calculation of the mean and extended high-energy tails can cause the mean Lorentz factor to be far-in-excess of the median Lorentz factor, therefore misrepresenting the bulk. Since the Debye screening length is very sensitive to a correct choice of temperature, we calculate the screening length that applies to a distribution function that well-represents the entire spectrum.

In our case, we fit to a Cauchy distribution (defined in the Methods section). The screening length which is derived from assuming this distribution takes the exact form

$$\lambda_{\mathrm{D}} = \lambda_{\mathrm{s}}\sqrt{\frac{\pi}{4}\Theta}\sqrt{\frac{\Theta^2-1}{\Theta^2-\frac{1}{2}}}\left[1-\frac{\sinh^{-1}\left(\sqrt{\Theta^2-1}\right)}{\Theta\sqrt{\Theta^2-1}\,(2\Theta^2-1)}\right]^{-1/2}, \tag{1}$$

which approximates to $\lambda_{\mathrm{D}} = \lambda_{\mathrm{s}}\sqrt{\frac{\pi}{4}\Theta}$ when $\Theta \gtrsim 1$. As shown in Supplementary Figure 6, this is only a slight modification from the screening length derived for a relativistic Maxwellian (Jüttner-Synge distribution), which is not an unexpected result since the two functions are approximately identical in the thermal bulk. It is reassuring that precise fitting of the extended tails is therefore not expected to be critical to the accuracy of the true screening length.

Since the screening length is derived assuming zero bulk motion, to obtain the appropriate pair temperature a Lorentz transformation is applied to the FLUKA-simulated momentum spectra to fit the data. The fitting of Cauchy and Jüttner-Synge distributions to the Lorentz-transformed momentum distributions is shown in Supplementary Figure 7. The fitted longitudinal temperature is $\Theta_{||} = 6.5$ and the transverse temperature is $\Theta_{\perp} = 3.5$.

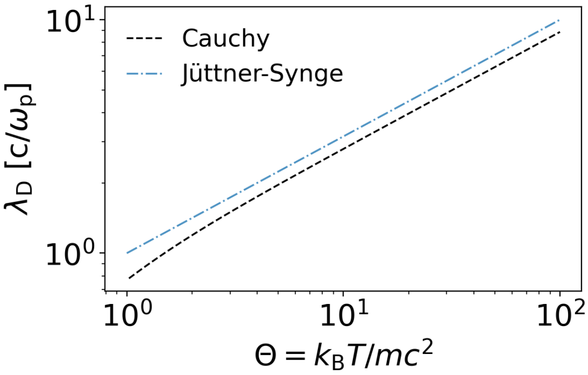

Supplementary Fig. 6: **Debye screening lengths derived for relativistic Cauchy and Jüttner-Synge distribution functions.** The screening lengths are plotted in units of collisionless skin depth λ_{s}. The similarity between the two screening lengths is clear, differing by only 12% when the normalized temperature $\Theta \gtrsim 1$.

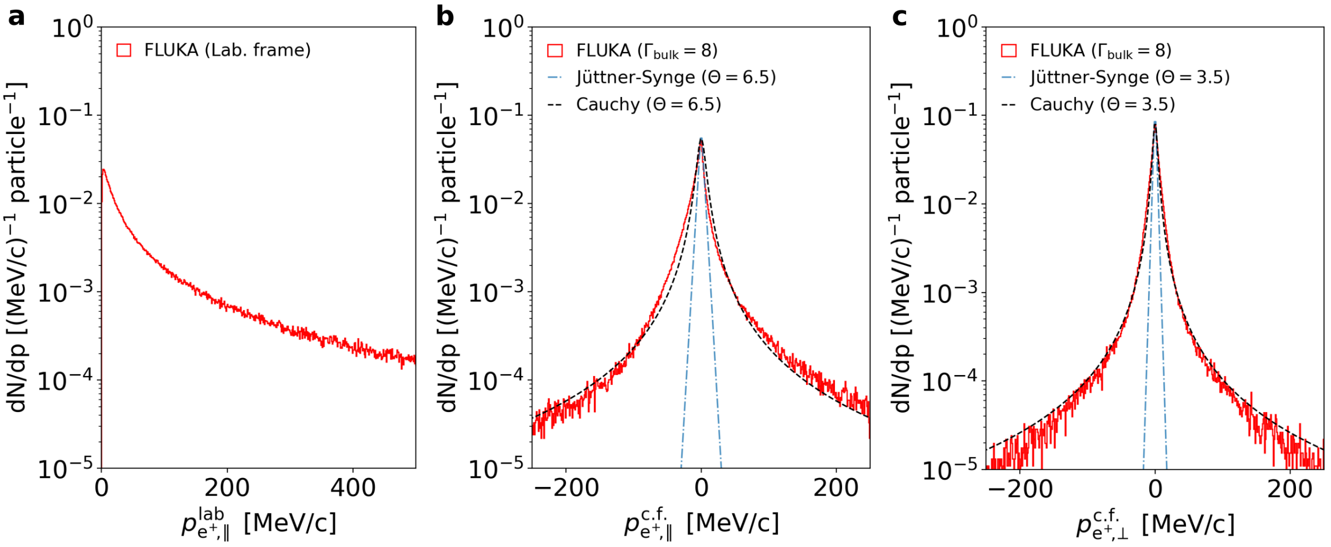

Supplementary Fig. 7: **Fitting the momentum distributions in the frame co-moving with the beam.** (a) The longitudinal momentum distribution of positrons in the laboratory frame, $p^{\mathrm{lab}}_{\mathrm{e+},\parallel}$ is obtained from FLUKA simulations (red). (b, c) A Lorentz transformation ($\Gamma_{\mathrm{bulk}} = 8$) is applied to the particle momentum vectors to obtain the longitudinal and transverse momentum distributions in the frame co-moving with the beam, $p^{\mathrm{c.f.}}_{\mathrm{e+},\parallel}$ and $p^{\mathrm{c.f.}}_{\mathrm{e+},\perp}$. The spectra shown in (b) and (c) correspond to 98.5% of the positrons. A Cauchy distribution provides a better fit to the spectrum than a relativistic Maxwellian (Jüttner-Synge) distribution. The obtained longitudinal temperature is $\Theta_{\parallel} = 6.5$ and the transverse temperature is $\Theta_{\perp} = 3.5$. A temperature $\Theta = 5.0 \pm 1.5$ is assumed to calculate the plasma screening length in the case of an isotropic Cauchy distribution.

6 Parameter scan of target material thicknesses using FLUKA simulations

The thickness of graphite and tantalum in the target were chosen based on the limitation of space in the experimental area and to achieve a low pair beam emittance, maximizing the measurable pair yield in downstream detectors. However, in the current design, a large fraction of the primary protons (40%) pass through the target without significant energy loss, and thicker targets may deplete the primary beam to a greater extent and generate a higher pair yield. An extensive parameter has been performed using FLUKA to investigate the pair densities achievable if different thicknesses of graphite and tantalum are used.

The thickness of graphite was simulated in steps of 100 mm up to 800 mm, whilst the thickness of tantalum was simulated up to 60 mm in steps of 10 mm. For each combination, the positron yield and peak density is obtained at the immediate rear of the target. A Gaussian beam profile ($\sigma = 1$ mm) is assumed for the primary beam. To obtain a smooth colourmap, the data points have been linearly interpolated and a Gaussian smoothing has been applied with kernel size equal to half the scanning interval (see Supplementary Figure 8). The results show that pair yields of 5×10^{13} and densities $8 \times 10^{12}\,\mathrm{cm}^{-3}$ may be achievable using thicker target materials (e.g. graphite thickness $= 400 - 600$ mm and tantalum thickness $= 25 - 30$ mm).

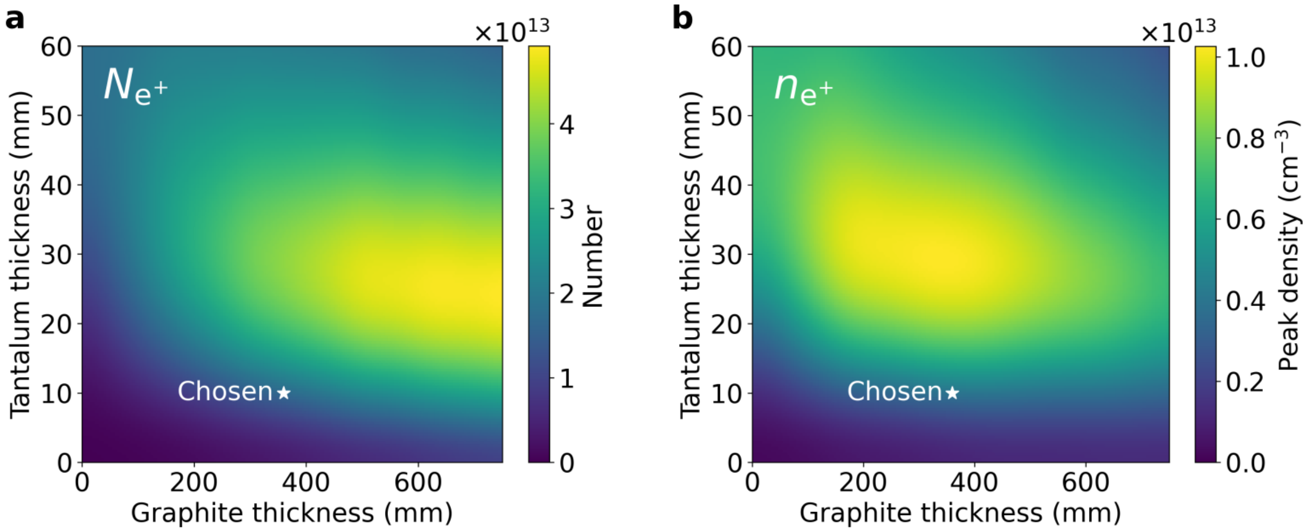

Supplementary Fig. 8: **Positron yield and peak density in a parameter scan of material thicknesses.** FLUKA Monte-carlo simulations of (a) the positron yield, and (b) the peak positron density, at the rear of the target when composed of a range of thicknesses of graphite (up to 800 mm) and tantalum (up to 60 mm). The primary beam is assumed to contain 3×10^{11} protons, and the results can be re-scaled for different primary intensities. The yield and density presented corresponds to positrons with kinetic energy greater than 1 MeV. The combination of graphite and tantalum thicknesses chosen for the experiment is shown by the star.

References

[1] Arrowsmith, C. D. *et al.* Generating ultradense pair beams using 400 GeV/c protons. *Phys. Rev. Res.* **3**, 023103 (2021), doi: https://doi.org/10.1103/PhysRevResearch.3.023103. Published under the Creative Commons Attribution 4.0 International license (https://creativecommons.org/licenses/by/4.0/).
